# Acceptability and fidelity of the multidomain ‘Brain Bootcamp’ dementia risk reduction program: a mixed-methods approach

**DOI:** 10.1186/s12889-025-21641-7

**Published:** 2025-02-14

**Authors:** Joyce Siette, Laura Dodds, Cristy Brooks, Kay Deckers, Sebastian Köhler, Christopher J. Armitage

**Affiliations:** 1 The MARCS Institute for Brain, Behaviour and Development, Western Sydney University, Westmead, NSW 2145 Australia; 2 NICM Institute, Western Sydney University, Westmead, NSW 2145 Australia; 3 Department of Psychiatry and Neuropsychology, School for Mental Health and Neuroscience, Alzheimer Centrum Limburg, Maastricht University, Maastricht, 6200 MD the Netherlands; 4 Manchester Centre for Health Psychology, University of Manchester, Manchester, M13 9PL UK; 5 NIHR Greater Manchester Patient Safety Research Collaboration, University of Manchester, Manchester, M13 9PL UK

**Keywords:** Dementia prevention, Outcome, Feasibility, Acceptability, Older adults, Lifestyle

## Abstract

**Background:**

Interventions targeting dementia prevention typically lack comprehensive exploration of feasibility, acceptability, and long-term translation factors prior to deployment. Our study aimed to explore the acceptability, fidelity and participants’ experiences with Brain Bootcamp, a multi-domain behaviour change intervention targeting reduced dementia risk and increased dementia risk factor awareness for older adults.

**Methods:**

Conducted in New South Wales, Australia, from January to August 2021, our concurrent single-group mixed-methods feasibility study involved post-intervention surveys and qualitative interviews with community-dwelling older adults. Descriptive statistics were used to assess acceptability of the methods, outcome measures, and fidelity to the program components. Thematic analysis of semi-structured interviews explored participant experiences, preferences, barriers, and recommendations.

**Results:**

Out of 853 enrolled participants, only 355 completed the program (41.6%). Among these participants, 79.1% agreed that the intervention improved their awareness of dementia risk factors, and 92.4% expressed intent to continue maintaining brain healthy behaviours post- program. Participants typically set 2–4 modifiable risk factor lifestyle goals, which were most often related to physical activity (83.7%). A majority (91.5%) successfully achieved at least one brain health goal. Qualitative analyses (*n* = 195) identified three overarching themes on the role of education on behaviour modification (i.e., the transformative role of the program in enhancing knowledge about dementia prevention and fostering behavioral modifications), psychological considerations (e.g., intrinsic versus extrinsic motivation on their engagement and perception of the program) and future directions (e.g., sustainability concerns and the need for tailored strategies for specific demographics).

**Conclusions:**

While Brain Bootcamp had low completion rates, those who completed the program reported high acceptability. Future refinements, incorporating targeted strategies and enhanced participant support and communication, will facilitate pragmatic initiatives.

**Clinical trial number:**

ACTRN12621000165886.

**Supplementary Information:**

The online version contains supplementary material available at 10.1186/s12889-025-21641-7.

## Introduction

Dementia is a condition where cognitive functioning declines beyond normal ageing and impacts on one’s ability to perform everyday social and working tasks [1]. This has ongoing impacts not only on the lives of people with dementia, their caregivers but also the wider community [1]. Exact numbers of the population living with dementia in Australia is currently unknown and likely underrepresented. In 2022, an estimated 400,000 to 459,000 Australians were diagnosed with dementia, and in the next 30 years this number is expected to double [2]. Despite extensive global research, there is no curative treatment available yet [3], which poses significant challenges for healthcare systems worldwide. As populations continue to age, the prevalence of dementia is expected to increase substantially, leading to a corresponding rise in healthcare costs [4].

Observational research strongly suggests that dementia can be potentially modifiable through behaviours [5]. These modifiable risk factors include lower levels of education, hearing loss, traumatic brain injury, hypertension, alcohol consumption, obesity, smoking, depression, social isolation, physical inactivity, diabetes, and exposure to air pollution [5]. However, results from randomised trials show mixed results [6]. For instance, the Dutch Prevention of Dementia by Intensive Vascular Care (preDIVA) found that a nurse-led intensive vascular care program delivered in primary care did not decrease risk of all-cause dementia, but lowered risk in a pre-planned subgroup analysis of people with untreated hypertension and the risk of non-Alzheimer’s disease dementia [7]. The Multi-domain Alzheimer Preventive Trial (MAPT) targeted nutrition, physical and cognitive activity through 3 individual or group consultations and found no differences in cognitive decline across the intervention groups in the 3 year trial period, but cognitive decline was less pronounced in participants with higher dementia risk as indicated by amyloid blood status [8]. The Finnish Geriatric Intervention Study to Prevent Cognitive Impairment and Disability (FINGER) tested the effect of a multidomain lifestyle and behaviour intervention of diet guidance, physical exercise, cognitive training and vascular risk management in 1260 older adults, and found a more favourable cognitive trajectory after two years in the intervention group [9]. The smaller scale SMARRT randomised clinical trial involving person-delivered health coaching also demonstrated improvements in cognitive scores and risk factor scores amongst older adults over two years [10].

Efforts to address modifiable dementia risk both nationally and internationally in the form of lifestyle programs are increasing in popularity [11]. Examples of trials either currently being designed or implemented include the Maintain Your Brain (MYB) trial [12], Japan-Multimodal Intervention Trial for Prevention of Dementia PRIME Tamba (J-MINT) [13], Canadian Therapeutic Platform Trial for Multidomain Interventions to Prevent Dementia (CAN-THUMBS UP) [14], the APPLE Tree program [15], the Body Brain Life trial [16], the LEISURE study [17] and the AgeWell.de [18] which target various dementia risk factors utilising numerous approaches such as physical resources [15], face-to-face lifestyle coach-delivered sessions [13, 16, 17], as well as m-health and web-based platforms [14, 15, 19] in an effort to educate and support lifestyle behaviours for brain health. Moreover, the collective efforts extend beyond the mentioned trials, with more ongoing trials aiming to contribute further insights and solutions to the complex challenge of dementia prevention [11].

Despite the above evidence to indicate that multidomain lifestyle interventions may be effective in promoting cognitive health and reducing dementia risk [6, 20, 21], these programs are often criticised for being time and resource consuming, too costly and require substantial caregiver support. Additionally, they tend to have limited generalisability as many trials involved highly selective populations that predominantly consisted of individuals from Anglo backgrounds. As a result, these interventions may struggle to achieve compliance or success when scaled for broader, more diverse, population-scale implementation [22, 23]. Furthermore, whilst acceptability and feasibility are both important aspects to consider in the design, evaluation and implementation of interventions are often not fully evaluated [24]. These aspects are critical to understanding the extent of anticipated cognitive and emotional responses of participants to the intervention as well as its appropriateness and practicality to scaling up of future programs [24].

Brain Bootcamp is an Australian multidomain dementia risk reduction program developed to increase dementia risk factor awareness and reduce dementia risk scores by addressing multiple modifiable risk factors for older adults. Following recommendations of the Medical Research Council (MRC) guidance for complex interventions [25], evaluations of program acceptability can support future program development. This paper thus aimed to explore the feasibility and acceptability of the Brain Bootcamp program using a mixed-methods approach.

## Methods

A brief methods overview is provided in accordance with guidance for reporting pilot trials with further details available in the published protocol [26] and the program’s effectiveness paper [27]. Ethics review was conducted and approved by Macquarie University Human Research Ethics Committee (reference number 9174). All participants provided written and verbal consent prior to entering the study. The project was registered in the clinical trial registry (ACTRN 12621000165886). There was no deviation from the published protocol.

### Trial design

Brain Bootcamp used a single-group pre-post study design and a mixed methods evaluation approach. Participants completed a baseline assessment, followed by the Brain Bootcamp intervention and a follow-up assessment three months later.

### Participants

Participants were recruited via widespread advertising strategies (see Siette et al., 2022 [26] for further information) from January 2021 to August 2021 across New South Wales, Australia. Community-dwelling adults aged 65 years or older who were literate in English and had access to the Internet or were able to access a hard copy of study documents were eligible for participation. Exclusion criteria were: self-reported active episode of major depression, existing diagnosis of dementia, individuals who were unable to provide informed consent, or ongoing involvement/enrolment in a behaviour change intervention.

### Intervention

The multidomain intervention aimed to address behaviour change related to physical activity, social engagement, healthy diets and cognitive activity to reduce dementia risk by adopting three behaviour change principles: education, environmental restructuring, and enablement, over a period of 3 months [28]. In order to achieve this, participants received a Brain Bootcamp box containing several resources designed to facilitate these changes. First, the box included personalised information based on the participants’ LIBRA dementia risk profile, a weighted compound score that assesses 12 modifiable risk and protective factors influencing dementia risk. This information was collected pre-program entry and personalised feedback detailing areas favourable for preserving brain health, aspects requiring lifestyle intervention and strengths in cognitive activity was provided in hard copy inside the box. The LIBRA index has been extensively validated and ranges from − 5.9 to 12.7, with higher scores indicating a greater risk of developing dementia [29–32]. Second, participants received an information booklet aimed at improving dementia literacy and promoting associated health-related behaviours, including advice on eating healthy and activities to be socially, mentally and physically active. This educational resource covered the topics on the significance of physical activity, balanced nutrition, cognitive stimulation, social engagement, and stress management in preserving optimal brain function and mitigating dementia risk. Third, the box contained four physical items designed to stimulate healthy brain habits within participants’ routines. This included a pedometer to encourage physical activity, brain teaser flash cards to target cognitive inactivity, a social calendar to address social isolation, and olive oil to promote healthier dietary choices. Lastly, participants were guided to set specific, measurable goals using resources in the educational booklet and social calendar to support their own personal and social development. They were encouraged to self-monitor their progress, with sections allocated for monthly goal setting. No minimum number of goals were prescribed, allowing participants to tailor their goal setting process to their individual needs and circumstances.

### Procedure

Upon enrolment, participants were requested to complete an initial online survey that included questions regarding dementia risk, dementia literacy, motivation to adopt lifestyle changes aimed at reducing dementia risk, mental health, quality of life, and social networks. Additional items assessed basic demographic information, such as age, gender, and educational level, as well as participants’ medical histories. Following the completion of the initial survey, participants received their box within seven days, which contained resources and materials to support their engagement over the subsequent three months (see Intervention above). Six weeks after receiving the box, the research team contacted participants via email to notify them of the halfway mark and further motivated participants to continue to engage in the program. The same survey was administered again at the end of the three month period. Participants also completed an impact evaluation survey on their experiences of the program and were invited to attend a semi-structured interview to share their perspectives. Participants did not receive any compensation for their involvement in the intervention.

### Materials

The feasibility and acceptability of the program was assessed by a mixed methods approach, including a quantitative survey and semi-structured interviews following the intervention. Sociodemographic information of participants was collected by the research team, in addition to records of recruitment and retention.

### Part 1: Impact evaluation survey

At the end of the program, participants were asked to complete a 48-item impact evaluation online survey derived from the overarching questions from the Australian Government impact evaluation framework [33] delivered via Qualtrics. The survey contained five domains including (i) overall experience (e.g., response to statements such as ‘*Brain Bootcamp increased my awareness about dementia risk factors*’ with a 5-point Likert scale of strongly agree to strongly disagree), (ii) open text responses on what was most liked and disliked about the program and recommendations for the future), (iii) recognition of the program’s call to action phrases (e.g., ‘*Your brain health is in your hands*’), (iv) item use and preference (e.g., frequency of use, preference order) and (v) evaluation for each risk factor (e.g., whether a physical/social/diet/cognitive goal was set, what the goal was, whether the participant was able to meet their set goal and how did the item support goal achievement). A copy of the survey is available in the Appendix for further reference.

### Part 2: Qualitative interviews

Semi-structured telephone interviews were conducted by JS (PhD, lead investigator, > 10 years qualitative experience) and LD (MPH, trial coordinator, > 5 years qualitative experience) between June and September 2021 to further understand the experience of participants enrolled in the program. They were recruited at the end of the trial period via email or telephone. Participants did not receive any incentive to be involved in the interview. Participants were informed that the research team aimed to understand their experiences and valued their perspectives. They were encouraged to openly share any biases, assumptions, or reasons related to the research topic. Participants may have been familiar with the research team prior to the interviews through their involvement in the program. This pre-existing relationship, established during the program, could have influenced their interactions and comfort level during the post-program interviews. However, this familiarity might also have encouraged them to provide more authentic and candid responses. We used a theoretical sampling approach to select participants (*N* = 195) based on their dementia risk score (high and low), gender (male and female), education (high and low), cultural background (English-speaking vs. non-English speaking) and socioeconomic status (high and low) for the interviews. This information was gathered concurrently with sociodemographic profiles obtained from the surveys. Given the existing demographic diversity in the sample at follow-up, we invited all participants to engage in the interviews. Interviews explored in-depth views of the information they received from their profile, whether they have accessed the resources in their Brain Bootcamp box, any health behaviour change goals, how they did/did not incorporate recommendations into their daily life, and their perception of the overall impact of the initiative. The interview guide is available in the Appendix and was pilot tested with a sample of older adults prior to this study. Interviews ranged from 45 to 70 min.

### Analysis

For quantitative data, descriptive statistics were applied to participant demographics and all outcome measures. Semi-structured interviews were audiotaped, transcribed verbatim using professional scribers and checked by the research team. Interview data was then initially analysed for content and themes that emerged between two researchers independently (JS and CB) and coded and categorised using NVivo (Version 17). Thematic data analysis was performed using an inductive approach. Although specific key research questions were answered and guided the analysis, open coding was applied with no pre-set codes; rather codes were developed from interpretation of the data and modified throughout the analysis as required. The general process of qualitative data extraction included the research team becoming independently familiar with the data, followed by initial coding of data to identify key meaningful themes and sub-themes relevant to the study objectives. The initial codes were then reviewed by both two members of the research team, re-considered with respect to coding and study objectives and then adapted as necessary to form emergent themes. Further refinement of emerging themes and sub-themes was performed based on subsequent discussions with the broader research team.

## Results

### Part 1: Survey results

Program participants’ characteristics have been reported elsewhere [27]. Briefly, 855 participants were initially enrolled in the program, however, only 355 participants (41.6%) completed the follow-up assessments. Statistical analyses showed significant demographic differences between program completers and non-completers. Completers were more likely to be women, born in an English-speaking country, and to possess higher levels of education (Table 1). Additionally, completers had significantly lower dementia risk scores (*p* < 0.016). Among those who completed the program, the mean age was 73.2 years (SD = 6.0), with a significant majority of participants (75.2%) identifying as female. The sample had high educational attainment, with 53% having advanced degrees. Furthermore, 88.5% of participants were born in English-speaking countries, and 79.8% resided in metropolitan areas.

**Table 1:** Summary of participant demographics by program and interview completion

	Enrolled and completed (*N* = 355) *N* (%)	Drop-out (*N* = 498) *N* (%)	*p*-value^a^	Completed interview (*N* = 162)	Did not participate in interview (*N* = 193) *N* (%)	*p*-value^b^
Gender						
Female	267 (75.2)	330 (66.3)	0.005*	126 (77.8)	141 (73.1)	0.306
Male	88 (24.8)	168 (33.7)		36 (22.2)	52 (26.9)	
Age (mean [SD])	73.2 [6.0]	73.4 [6.2]	0.644	72.5 [5.3]	73.8 [6.6]	0.138
65–69	122 (34.4)	154 (30.7)	0.154	61 (37.7)	61 (31.6)	0.059
70–79	175 (49.3)	269 (53.7)		82 (50.6)	93 (48.2)	
80+	55 (16.3)	88 (15.6)		19 (11.7)	39 (20.2)	
Country of birth						
English-speaking country	314 (88.5)	405 (81.0)	0.003*	146 (90.1)	168 (87.0)	0.367
Non-English speaking country	41 (11.5)	95 (19.0)		16 (9.9)	25 (13.0)	
Education						
Low	96 (27.0)	197 (40.1)	< 0.001*	42 (25.9)	54 (28.0)	0.517
Intermediate	71 (20.0)	105 (21.4)		31 (19.1)	40 (20.7)	
High	188 (53.0)	189 (38.5)		89 (54.9)	99 (51.3)	
Socioeconomic status (quintile)						
1 (lowest)	17 (4.9)	26 (5.8)	0.107	6 (3.7)	11 (5.7)	0.097
2	51 (14.7)	74 (16.6)		19 (11.7)	32 (16.6)	
3	51 (14.7)	87 (19.6)		25 (15.4)	26 (13.5)	
4	38 (11.0)	57 (12.8)		16 (9.9)	22 (11.4)	
5 (highest)	190 (54.8)	201 (45.2)		95 (58.6)	95 (49.2)	
Locality						
Metropolitan	281 (79.8)	346 (76.0)	0.401	133 (82.1)	148 (76.7)	0.234
Regional	71 (21.2)	109 (23.9)		28 (17.3)	43 (22.3)	

^a^ Significance level set at < 0.05 for comparisons between the enrolled sample and the drop out sample

^b^ Significance level set at < 0.05 for comparison between the sample who participated in the interviews compared to those that did not

### Acceptability

A significant majority of participants agreed or strongly agreed that Brain Bootcamp increased their awareness about dementia risk factors (79.1%), and a substantial proportion reported learning much about brain healthy behaviours (75.3%) (Table 2). The personalised brain health profile was perceived as useful by a majority (72.4%) of respondents. While most felt they were provided with the right amount of resources to change their lifestyle (62.0%), there was room for improvement. Nearly half of respondents believed that Brain Bootcamp improved their brain health (48.1%) and the willingness to continue maintaining brain healthy behaviors was remarkably high, with 92.4% expressing their commitment. Overall satisfaction with the Brain Bootcamp initiative was positive, with 84.8% of participants reported being satisfied.

**Table 2:** Proportion of respondents’ agreement with feasibility and acceptability statements of the program (*n* = 355)

Statement	Agree / Strongly Agree *N* (%)	Neutral *N* (%)	Disagree / Strongly Disagree *N* (%)	Missing *N* (%)
Brain Bootcamp increased my awareness about dementia risk factors.	281 (79.1)	49 (13.8)	12 (3.4)	13 (3.7)
I learnt a lot about brain healthy behaviours.	267 (75.3)	54 (15.2)	15 (4.3)	19 (5.4)
I found my personalised brain health profile useful.	257 (72.4)	71 (20.0)	12 (3.4)	15 (4.2)
I was provided with the right amount of resources to change my lifestyle.	220 (62.0)	92 (25.9)	27 (7.6)	16 (4.5)
Brain Bootcamp improved my brain health.	171 (48.1)	147 (41.4)	21 (5.9)	16 (4.5)
I will continue to maintain my brain healthy behaviours.	328 (92.4)	14 (3.9)	5 (1.4)	8 (2.3)
Overall, I was satisfied with the Brain Bootcamp initiative.	301 (84.8)	31 (8.7)	12 (3.3)	11 (3.1)

**Fig. 1:**
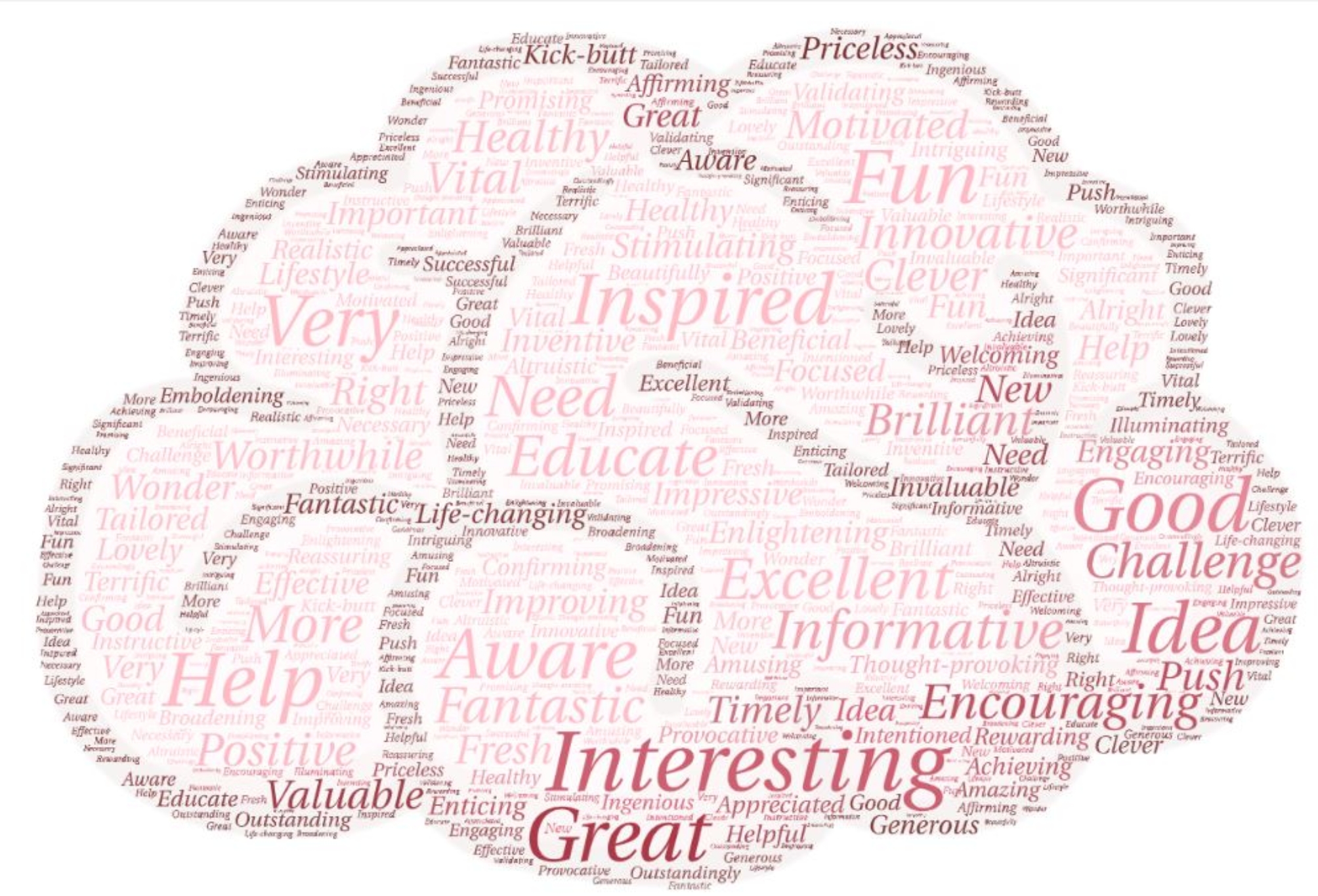
Word cloud of responses for a one-word description of the participant’s experience (*n* = 355)

Figure 1 presents a word cloud of the one-word responses provided by participants in the survey when asked to describe their experience of the program. The word cloud shows that several words were more prominent among participants, including, ‘Interesting’, ‘Fun’, ‘Great’, ‘Good’, ‘Idea’, ‘Inspired’, ‘Help’, ‘Aware’, ‘Informative’, ‘Encouraging’, ‘Educate’, ‘Valuable’ and ‘Challenge’.

### Goal setting

Table 3 shows the type of health behaviour goal set by respondents. Most respondents indicated they had set a goal (93.8%). There were two distinct goal categories where ‘general’ goals contained broad and overarching recommendations that did not specify detailed actions (e.g., “be more active), and ‘specific’ goals were more detailed and precise recommendations that had detailed guidance on particular actions, often with clear parameters or requirements (e.g., “exercise 30 minutes a day”). For general goals, diet (85.7%) and cognitive activity (81.8%) were most reported. For specific goals, most participants identified physical activity (67.9%) as their main goal. The majority of respondents confirmed that they had been successful in achieving any one of the health goals they had set for themselves (91.5%) with only 8.5% of respondents not achieving any of the set goals. Physical activity goals were commonly achieved (71.5%) whilst cognitive activity was the least achieved (56.4%).

**Table 3:** Summary of set and achieved goals by risk factor

	Overall	Physical activity	Social activity	Cognitive activity	Diet
Goal set					
Yes	333 (93.8)	297 (83.7)	240 (67.6)	223 (62.8)	246 (69.3)
No	22 (6.2)	44 (12.4)	97 (27.3)	110 (31.0)	91 (25.6)
Number of goals set					
0	22 (6.2)	-	-	-	-
1	23 (6.5)	-	-	-	-
2	77 (21.7)	-	-	-	-
3	103 (29.0)	-	-	-	-
4	130 (36.6)	-	-	-	-
Goal type set*					
General	-	88 (32.1)	153 (75.5)	171 (81.8)	191 (85.7)
Specific	-	186 (67.9)	58 (27.5)	38 (19.2)	32 (14.3)
Goal attainment					
Yes	325 (91.5)	254 (71.5)	219 (61.7)	201 (56.6)	211 (59.4)
No	30 (8.5)	101 (28.5)	136 (38.3)	154 (43.4)	144 (40.6)
Number of goals attained					
0	30 (8.5)	-	-	-	-
1	42 (11.8)	-	-	-	-
2	96 (27.0)	-	-	-	-
3	97 (27.3)	-	-	-	-
4	90 (25.4)	-	-	-	-

*General included statements such as “be more active” vs. specific “exercise 30 mins a day”

The most prevalent number of goals set was four, constituting 36.6% of respondents, followed by three goals at 29.0%, and two goals at 21.7%. The attainment of two or three goals was almost equal, accounting for 27.0% and 27.3%, respectively. A quarter of respondents achieved four goals (25.4%).

In terms of goal setting and attainment, distinct demographic variations emerged (Tables 4 and 5). Females exhibited a higher propensity for setting goals compared to males (*p* = 0.043), particularly in the domains of social (*p* = 0.005) and cognitive goals (*p* = 0.003). Moreover, females were more successful in achieving both social and cognitive goals (both *p* < 0.05). Participants born in English-speaking countries demonstrated a greater inclination toward goal setting compared to those born in non-English speaking countries (*p* = 0.001), with a specific emphasis on physical activity, social activity, and cognitive goals (all ps < 0.05). English-speaking individuals also exhibited a higher goal attainment rate (50.3% vs. 36%) and achieved their social and cognitive activity goals (all ps < 0.05) compared to individuals born in non-English speaking countries. Furthermore, individuals with higher education attainment set more goals (59.2% vs. 40.3%) compared to individuals with lower educational attainment. Compared to individuals with higher educational attainment, individuals with lower educational attainment set more physical activity goals but fewer social and cognitive goals. In terms of goal attainment, individuals with higher educational levels achieved their overall goals (*p* < 0.001) and were successful in reaching their physical activity, social activity, cognitive activity, and diet goals (all ps < 0.05), and in achieving a greater number of goals compared to people with lower education (*p* < 0.001). There were no significant differences in goal setting or attainment observed based on age, socioeconomic status, or locality.

**Table 4:** Association of demographic factors with goals set and goals achieved

	Gender			Age				Country of birth			Locality		
	Female N (%)	Male N (%)	*p*-value	65–69 N (%)	70–79 N (%)	80+ N (%)	*p*-value	English-speaking N (%)	Non-English speaking N (%)	*p*-value	Metropolitan N (%)	Regional N (%)	*p*-value
Goal set													
Yes	311 (52.1)	114 (44.5)	0.043*	147 (53.3)	213 (48.0)	65 (47.8)	0.345	374 (52.0)	50 (36.8)	0.001*	332 (53.0)	88 (48.9)	0.336
No	286 (47.9)	142 (55.5)		129 (46.7)	231 (52.0)	71 (52.2)		345 (48.0)	86 (63.2)		295 (47.0)	92 (51.1)	
Goal set (specific)													
Physical activity: Yes	270 (45.2)	102 (39.8)	0.084	124 (44.9)	192 (43.2)	56 (41.2)	0.764	328 (45.6)	43 (31.6)	0.003*	291 (46.4)	77 (42.8)	0.397
Physical activity: No	327 (54.8)	154 (60.2)		152 (55.1)	252 (56.8)	80 (58.8)		391 (54.4)	93 (68.4)		336 (53.6)	103 (57.2)	
Social activity: Yes	228 (38.2)	72 (28.1)	0.005*	104 (37.7)	147 (33.1)	49 (36.0)	0.442	269 (37.4)	31 (22.8)	< 0.001*	230 (36.7)	68 (37.8)	0.793
Social activity: No	369 (61.8)	184 (71.9)		172 (62.3)	297 (66.9)	87 (64.0)		450 (62.6)	105 (77.2)		397 (63.3)	112 (62.2)	
Cognitive activity: Yes	217 (36.3)	66 (25.8)	0.003*	100 (36.2)	142 (32.0)	41 (30.1)	0.366	250 (34.8)	33 (24.3)	0.017*	222 (35.4)	58 (32.2)	0.477
Cognitive activity: No	380 (63.7)	190 (74.2)		176 (63.8)	302 (68.0)	95 (69.9)		469 (65.2)	103 (75.7)		405 (64.6)	122 (67.8)	
Diet: Yes	233 (39.0)	83 (32.4)	0.075	114 (41.3)	159 (35.8)	43 (31.6)	0.125	274 (38.1)	42 (30.9)	0.121	241 (38.4)	71 (39.4)	0.862
Diet: No	364 (61.0)	173 (67.6)		162 (58.7)	285 (64.2)	93 (68.4)		445 (61.9)	94 (69.1)		386 (61.6)	109 (60.6)	
Number of goals set													
0	286 (47.9)	142 (55.5)	0.079	129 (46.7)	231 (52.0)	71 (52.2)	0.755	345 (48.0)	86 (63.2)	0.023*	295 (47.0)	92 (51.1)	0.402
1	20 (3.4)	13 (5.1)		11 (4.0)	15 (3.4)	7 (5.1)		29 (4.0)	3 (2.2)		28 (4.5)	4 (2.2)	
2	69 (11.6)	30 (11.7)		36 (13.0)	47 (10.6)	16 (11.8)		87 (12.1)	12 (8.8)		76 (12.1)	21 (11.7)	
3	98 (16.4)	34 (13.3)		41 (14.9)	73 (16.4)	18 (13.2)		114 (15.9)	18 (13.2)		108 (17.2)	24 (13.3)	
4	124 (20.8)	37 (14.5)		59 (21.4)	78 (17.6)	24 (17.6)		144 (20.0)	17 (12.5)		120 (19.1)	39 (21.7)	
Goal attainment													
Overall: Yes	301 (50.4)	111 (43.4)	0.059	143 (51.8)	207 (46.6)	62 (45.6)	0.324	362 (50.3)	49 (36.0)	0.002*	322 (51.4)	85 (47.2)	0.328
Overall: No	296 (49.6)	145 (56.6)		133 (48.2)	237 (53.4)	74 (54.4)		357 (49.7)	87 (64.0)		305 (48.6)	95 (52.8)	
Physical activity: Yes	227 (38.0)	84 (32.8)	0.163	110 (39.9)	159 (35.8)	42 (30.9)	0.194	270 (37.6)	40 (29.4)	0.080	244 (38.9)	63 (35.0)	0.384
Physical activity: No	370 (62.0)	172 (67.2)		166 (60.1)	285 (64.2)	94 (69.1)		449 (62.4)	96 (70.6)		383 (61.1)	117 (65.0)	
Social activity: Yes	201 (33.7)	64 (25.0)	0.012*	92 (33.3)	133 (30.0)	40 (29.4)	0.580	237 (33.0)	28 (20.6)	0.004*	206 (32.9)	57 (31.7)	0.787
Social activity: No	396 (66.3)	192 (75.0)		184 (66.7)	311 (70.0)	96 (70.6)		482 (67.0)	108 (79.4)		421 (67.1)	123 (68.3)	
Cognitive activity: Yes	194 (32.5)	60 (23.4)	0.009*	87 (31.5)	134 (30.2)	33 (24.3)	0.299	226 (31.4)	28 (20.6)	0.011*	200 (31.9)	51 (28.3)	0.411
Cognitive activity: No	403 (67.5)	196 (76.6)		189 (68.5)	310 (69.8)	103 (75.7)		493 (68.6)	108 (79.4)		427 (68.1)	129 (71.7)	
Diet: Yes	196 (32.8)	74 (28.9)	0.296	95 (34.4)	135 (30.4)	40 (29.4)	0.447	235 (32.7)	35 (25.7)	0.131	208 (33.2)	59 (32.8)	0.495
Diet: No	401 (67.2)	182 (71.1)		181 (65.6)	309 (69.6)	96 (70.6)		484 (67.3)	101 (74.3)		419 (66.8)	121 (67.2)	
Number of goals attained													
0	296 (49.6)	145 (56.6)	0.124	133 (48.2)	237 (53.4)	74 (54.4)	0.140	357 (49.7)	87 (64.0)	0.028*	305 (48.6)	95 (52.8)	0.758
1	39 (6.5)	23 (9.0)		21 (7.6)	28 (6.3)	13 (9.6)		52 (7.2)	9 (6.6)		50 (8.0)	10 (5.6)	
2	91 (15.2)	32 (12.5)		45 (16.3)	56 (12.6)	22 (16.2)		112 (15.6)	11 (8.1)		94 (15.0)	28 (15.6)	
3	87 (14.6)	29 (11.3)		35 (12.7)	71 (16.0)	10 (7.4)		100 (13.9)	16 (11.8)		92 (14.7)	24 (13.3)	
4	84 (14.1)	27 (10.5)		42 (15.2)	52 (11.7)	17 (12.5)		98 (13.6)	13 (9.6)		86 (13.7)	23 (12.8)	

**Table 5:** Association of socioeconomic status and education with goals set and goals achieved

	Socioeconomic status						Education			
	1 (low) N (%)	2 N (%)	3 N (%)	4 N (%)	5 (high) N (%)	*p*-value	Low N (%)	Moderate N (%)	High N (%)	*p*-value
Goal set										
Yes	21 (48.8)	60 (48.0)	66 (47.8)	49 (51.6)	222 (56.8)	0.255	118 (40.3)	84 (47.7)	223 (59.2)	< 0.001*
No	22 (51.2)	65 (52.0)	72 (52.2)	46 (48.4)	149 (43.2)		175 (59.7)	92 (52.3)	154 (40.8)	
Goal set (specific)										
Physical activity: Yes	19 (44.2)	54 (43.2)	56 (40.6)	40 (42.1)	198 (50.6)	0.198	192 (65.5)	104 (59.1)	178 (47.2)	< 0.001*
Physical activity: No	24 (55.8)	71 (56.8)	82 (59.4)	55 (57.9)	193 (49.4)		101 (34.5)	72 (70.9)	199 (52.8)	
Social activity: Yes	15 (34.9)	41 (32.8)	48 (34.8)	33 (34.7)	158 (40.4)	0.486	83 (28.3)	54 (60.7)	163 (43.2)	< 0.001*
Social activity: No	28 (65.1)	84 (67.2)	90 (65.2)	62 (65.3)	233 (59.6)		210 (71.7)	122 (69.3)	214 (58.8)	
Cognitive activity: Yes	13 (30.2)	42 (33.6)	46 (33.3)	34 (35.8)	144 (36.8)	0.864	76 (25.9)	55 (31.3)	152 (40.3)	< 0.001*
Cognitive activity: No	30 (69.8)	83 (66.4)	92 (66.7)	61 (64.2)	247 (63.2)		217 (74.1)	121 (68.8)	255 (59.7)	
Diet: Yes	19 (44.2)	47 (37.6)	51 (37.0)	34 (35.8)	160 (40.9)	0.769	98 (33.4)	61 (34.7)	157 (41.6)	0.066
Diet: No	24 (55.8)	78 (62.4)	87 (63.0)	61 (64.2)	231 (59.1)		195 (66.6)	115 (65.3)	220 (58.4)	
Number of goals set										
0	22 (51.2)	65 (52.0)	72 (52.2)	46 (48.4)	169 (43.2)	0.768	175 (59.7)	92 (52.3)	154 (40.8)	< 0.001*
1	1 (2.3)	3 (2.4)	3 (2.2)	7 (7.4)	17 (4.3)		7 (2.4)	6 (3.4)	20 (5.3)	
2	4 (9.3)	15 (12.0)	16 (11.6)	11 (11.6)	52 (13.3)		31 (10.6)	22 (12.5)	46 (12.2)	
3	7 (16.3)	17 (16.6)	22 (15.9)	12 (12.6)	73 (18.7)		31 (10.6)	32 (18.2)	69 (18.3)	
4	9 (20.9)	25 (20.0)	25 (18.1)	19 (20.0)	80 (20.5)		49 (16.7)	24 (13.6)	88 (23.3)	
Goal attainment										
Overall: Yes	20 (46.5)	58 (46.4)	65 (47.1)	46 (48.4)	217 (55.5)	0.227	114 (38.9)	80 (45.5)	218 (57.8)	< 0.001*
Overall: No	23 (53.5)	67 (53.6)	73 (52.9)	49 (51.6)	174 (44.5)		179 (61.1)	96 (54.5)	159 (42.2)	
Physical activity: Yes	15 (34.9)	44 (35.2)	43 (31.2)	34 (35.8)	170 (43.5)	0.080	81 (27.6)	59 (33.5)	171 (45.4)	< 0.001*
Physical activity: No	28 (65.1)	81 (64.8)	95 (68.8)	61 (64.2)	221 (56.5)		212 (72.4)	117 (66.5)	206 (54.6)	
Social activity: Yes	13 (30.2)	34 (27.2)	42 (30.4)	27 (28.4)	146 (37.3)	0.154	74 (25.3)	49 (27.8)	142 (37.7)	0.001*
Social activity: No	30 (69.8)	91 (72.8)	96 (69.6)	68 (71.6)	245 (62.7)		219 (74.7)	127 (72.2)	235 (62.3)	
Cognitive activity: Yes	12 (27.9)	36 (28.8)	43 (31.2)	28 (29.5)	131 (33.5)	0.810	68 (23.2)	48 (27.3)	138 (36.6)	< 0.001*
Cognitive activity: No	31 (72.1)	89 (71.2)	95 (68.8)	67 (70.5)	260 (66.5)		225 (76.8)	128 (72.7)	239 (64.3)	
Diet: Yes	13 (30.2)	41 (32.8)	44 (31.9)	27 (28.4)	142 (36.3)	0.581	79 (27.0)	53 (30.1)	138 (36.6)	0.025*
Diet: No	30 (69.8)	84 (67.2)	94 (68.1)	68 (71.6)	249 (63.7)		214 (73.0)	123 (69.9)	239 (63.4)	
Number of goals attained										
0	23 (53.5)	67 (53.6)	73 (52.9)	49 (51.6)	174 (44.5)	0.300	179 (61.1)	96 (54.5)	159 (42.2)	< 0.001*
1	2 (4.7)	9 (7.2)	7 (5.1)	13 (13.7)	28 (7.2)		20 (6.8)	11 (6.3)	31 (8.2)	
2	8 (18.6)	18 (14.4)	24 (17.4)	10 (10.5)	63 (16.1)		36 (12.3)	25 (14.2)	62 (16.4)	
3	5 (11.6)	14 (11.2)	19 (13.8)	9 (9.5)	69 (17.6)		22 (7.5)	28 (15.9)	66 (17.5)	
4	5 (11.6)	17 (13.6)	15 (10.9)	14 (14.7)	57 (14.6)		36 (12.3)	16 (9.1)	59 (15.6)	

### Item usage

The most frequently used items from the Brain Bootcamp box were olive oil and balsamic vinegar, and the pedometer, with 35.5% and 34.4% of respondents using these items on a daily basis, respectively (Table 6). The least used item was the social calendar, with 42.3% of respondents choosing not to use this item. In terms of frequency, the brain activity cards were commonly used less than once per month (30.4%), with the education booklet also being used at a similar frequency (47%). There was a definitive spread of use of box items by the respondents, as use of different items varied across at least once per week, less than once a week and less than once a month (Table 5).

**Table 6:** Proportion of respondents’ that used items in the Brain Bootcamp box (*n* = 355)

Item	< Once a month	< Once a week	≥ Once a week	Did not use	Every day (or close to)
Social calendar	41 (11.5)	29 (8.2)	51 (14.4)	150 (42.3)	63 (17.7)
Olive oil and balsamic vinegar	31 (8.7)	38 (10.7)	115 (32.4)	32 (9.0)	126 (35.5)
Brain activity cards	108 (30.4)	83 (23.4)	68 (19.2)	61 (17.2)	21 (5.9)
Pedometer	33 (9.3)	25 (7.0)	28 (7.9)	137 (38.6)	122 (34.4)
Education booklet	167 (47.0)	63 (17.7)	48 (13.5)	52 (14.6)	9 (2.5)

### Part 2: Interviews

**Fig. 2:**
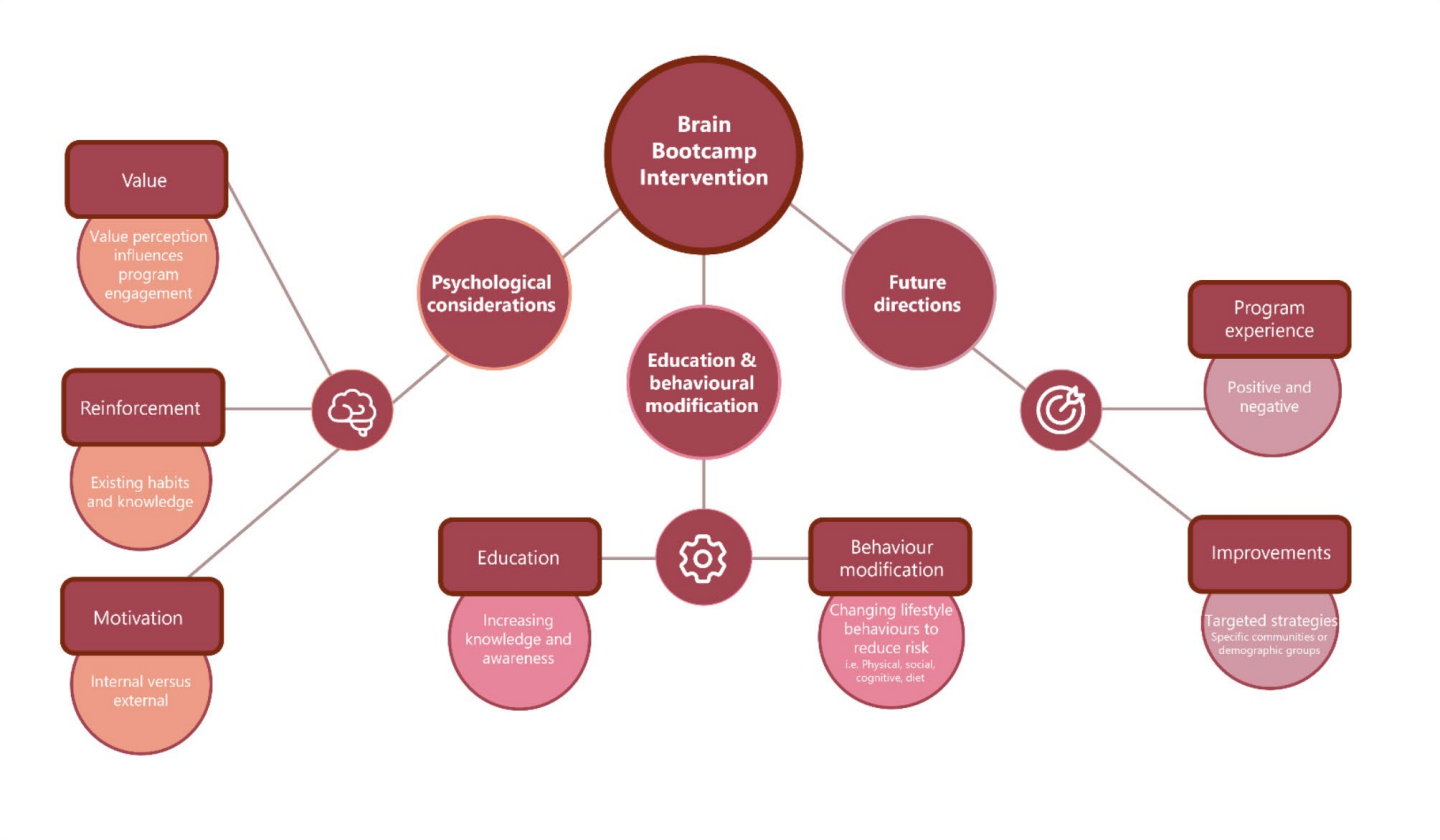
Diagrammatic representation of emerging themes and sub-themes from qualitative analysis of semi-structured interviews

All program completers were invited to attend a semi-structured interview. Of these, over half of program completers participated (162/355, 54.9%). Table 1 shows a summary of their demographic characteristics. There were no significant demographic differences between participants who completed the interview compared to those who did not (ps > 0.05). Thematic analysis identified three key themes and eight sub-themes (Fig. 2).

### Theme 1: Education and behavioural modification

#### Education

Participants consistently highlighted how the program increased their knowledge and awareness of dementia.

*“I didn’t realise that there were so many other medical conditions that might have an impact on dementia.” (P516)*.

Participants found the program to be a valuable resource for gaining knowledge, particularly on dementia risk factors, and methods for reducing the likelihood of developing the condition. Real-life experiences with friends or family affected by dementia, coupled with personal exposure to memory changes, fueled participants’ interest in the program.

*“I found it informative. I also thought it was good that it sort of breaks the silence” (P35)*.

The program’s impact extended beyond traditional knowledge and prompted thought-provoking considerations of dementia risk factors and healthy habits.

*“…I think it made me more aware of at any age that you can make things better for yourself.” (P493)*.

Some expressed they had knowledge of dementia prior to Brain Bootcamp but did not necessarily use it. Others felt it corrected some previously inaccurate knowledge in their thinking.

*“I’ve done some research about my mum*,* and I got a shock to think that I got answers that were wrong.” (P501)*.

#### Behavioural modification

Many participants noted that the program encouraged them to contemplate their behaviours and served as a catalyst for both thought and lifestyle changes to mitigate the risk of developing dementia.

*“It alerted me to stuff I needed to do to improve or maintain my brain health. It inspired me to look up different recipes. It suggested to me that I needed to exercise much more. So*,* I bought a dog and when she is old enough*,* we will be going for long walks.” (P70)*.

Several participants indicated alterations to their current lifestyle behaviours following the program. Specifically, participants mentioned changes in certain behaviours, such as dietary adjustments, increased engagement in brain-stimulating activities, or more regular exercise. This often translated into the establishment of new routines or the development of personalised plans.

*“It did make a big difference to my routine. I’ve got to get up and do the cards and the walking and the plan. I had a plan in place.” (P24)*.

Others reported a broader awareness of their lifestyle choices and expressed an increased consciousness of their daily activities due to Brain Bootcamp. For instance, some mentioned that it reinforced existing practices or assisted in refining their lifestyle choices rather than necessitating a complete shift in direction.

*“It[the program] improved it. It wasn’t a redirection it was more a refinement of things that I should be really working on like social connection.” (P285)*.

In general, participants described how the program encouraged behaviour change through the resources in the program.

*“I think that the cards were very good and very challenging.” (P172)*.

Some individuals preferred the practical tools, such as handouts, where others preferred the educational materials to increase knowledge.

*“I think just reading through that booklet*,* I kind of read through it a couple of times and each time I read through it*,* I would pick something else out.” (P501)*.

There was an additional observation emphasising the need for a well-structured plan when undertaking lifestyle changes. Several participants expressed that the information and resources provided by Brain Bootcamp presented a challenge. Given their limited prior knowledge about dementia, participants found themselves compelled to reassess their lifestyle choices and implement improvements.

*“It challenged me to look at my risk factors. I was a bit vague about a few of them.” (P598)*.

### Theme 2: Psychological considerations

#### Internal versus external motivation

A distinct contrast emerged among participants regarding internally driven motivational levels and external sources of motivation. Participants relying on external motivation highlighted the value of the structured system provided by the program and found enjoyment in its systematic approach. In contrast, others derived internal motivation from guidance and support and appreciated the non-compulsory nature of the program.

*“It was just good to have a system. It was good to get information about what worked and what was good for your brain.” (P187)*.

They further valued the reassurance and confirmation of what they were doing to know they were on the right track.

*“All the strategies that you explained in the booklet were very helpful.” (P257)*.

Others mentioned the positive perception of the program’s existence and recognised it as a scientific initiative focused on understanding and improving brain health.

*“I liked the fact that it’s actually happening*,* that there is research being done about brain fitness.” (P219)*.

Some participants found the inclusion of physical measures, such as tracking through the pedometer, helpful for visualising their progress and engagement.

*“I thought the tracking device was very useful because if you wore it every day it forced you to understand how many steps you were taking.” (P268)*.

Others derived internal motivation from the guidance and support provided by the program, employing self-help and reflection without feeling compelled to participate. The sense of routine offered reassurance and a feeling of not being alone.

*“I think it’s got value. It makes you reflect on what you do.” (P54)*.

There was an overlap between internal motivation and psychological benefits from the program. Participants reflected on mental gains, expressing gratitude for confirmation that they were on the right path. The sense of routine offered reassurance and a feeling of camaraderie on their health journey.

“*It gave me a guideline of things that I should do. I liked where they gave you a synopsis of where you are at now*,* and the things that I needed to work on.” (P585)*.

However, some participants reported not continuing with the suggestions from the program or experiencing no noticeable differences. Many of these participants expressed that it was a conscious choice not to continue, feeling they were already leading a healthy lifestyle and did not see the need for further changes.

“*I was already doing exercise and I’ve kept that up.” (P187)*.

Some acknowledged their lack of engagement with the program and reflected that it was the individual’s responsibility for making changes.

*“You have to be self-motivated for it to work*,* and I find that really hard.” (P26)*.

#### Program value and engagement

The degree of engagement in the program was influenced by the perceived value and motivation, whether stemming from internal or external sources. Participants who intrinsically valued the program and were consequently self-motivated demonstrated a heightened sense of purpose, personal value, contribution, and overall appreciation for Brain Bootcamp.

*“It probably encouraged me to get out and go for a walk along the beach in the morning. Because I firmly believe all good information is useful information and then it’s up to you how you utilise it.” (P617)*.

The feeling of the overall concept of the Brain Bootcamp was generally positive, as participants acknowledged it made them think differently. There was feedback on it being a good program and that it was making a difference. Participants expressed a sense of enjoyment and clarity of the information provided. They thought the nature of the program was valuable.

*“I thought it was a very good initiative.” (P92)*.

#### Reinforcement of existing habits and knowledge

There was a recurring theme of reinforcement of existing knowledge and behaviours among research participants. Participants found the program increased their confidence and reaffirmed their knowledge of dementia.

*“I think so*,* it’s given me more confidences.” (P514)*.

Participants in the program found it beneficial as it affirmed their existing knowledge of dementia risk factors. Moreover, they appreciated that the program was supportive of risk reduction rather than solely emphasising behaviour change.

*“I believe that can do things to limit my risk of getting it [dementia].” (P69)*.

They felt it reinforced their existing habits around lifestyle behaviours by encouraging healthy lifestyle choices, providing reminders and increasing their knowledge and understanding of how these factors relate to dementia risk. It made them conscious of their health, and many spoke about more confidence, reaffirmed/confirmed knowledge, and beliefs.

*“…well it confirmed what I thought you know. A lot of what I thought*,* and it was good and reassuring to know I was sort of on the right track.” (P582)*.

### Theme 3: Future directions

#### Mixed responses on program experience

Responses to the program experience varied, and opinions differed on which audience would derive the most benefit. The majority of participants viewed the program very positively, offering no negative comments. Their feedback was overwhelmingly favourable, expressing satisfaction with every aspect of the program.

*“There was nothing I particularly didn’t like.” (P128)*.

*“As a public health program I thought it was brilliant.” (P581)*.

Participants offered varied feedback on the program, using descriptors such as ‘interesting’, ‘surprising’, ‘helpful’, ‘challenging’, ‘reinforcing’, ‘visual’, ‘highlighting areas to improve’, ‘summarizing’, ‘awareness-raising’, and ‘simple’. They appreciated the program’s ability to stimulate their thinking and prompt contemplation on various aspects.

*“The whole concept of it was great in my opinion. I liked the fact that it was just getting me thinking and looking at different things.” (P219)*.

However, certain participants found aspects of the program unrealistic or not useful, considering certain elements too difficult, irrelevant, or lacking novelty. Concerns were raised about the absence of feedback, including insufficient depth of content.

*“See*,* what I did not like is that there is no involvement between me and them. They just sent me a box and expect me to follow it*,* end of the story.” (P277)*.

Additionally, some participants felt that the program did not effectively address their fears regarding the condition, particularly among those with a higher risk of dementia.

*“The chances of me getting it [Alzheimer’s] are extremely high and I didn’t think it covered*,* if you would like*,* easing the stress of that a bit.” (P219)*.

There was also a recurring issue about a lack of consideration for sustainability, such as excessive and non-recyclable packaging.

*“I think maybe the box that it came in was a bit over the top. None of it I could really recycle which annoys me.” (P634)*.

#### Suggestions for future programs

Participants expressed a clear consensus that the program could be enhanced through targeted strategies in the future. Opinions varied regarding the most relevant audience to target, with suggestions ranging from specific demographics (ethnic groups, retirees) to those with exposure to dementia. There were diverse perspectives on age groups that would benefit the most, from specific categories to more general groups like older adults.

*“Probably*,* it might be quite good for people who have recently retired. Because you know*,* they suddenly lose a lot of their social contacts*,* they lose the exercise of getting up and going to work every day.” (P635)*.

*“Oh*,* I think anybody really because it’s all good stuff you know.” (P493)*.

Another perspective highlighted the importance of the motivational aspect, suggesting that the program would be beneficial for those needing personal encouragement and lacking healthy habits.

*“I think it would make a difference to people who are not motivated to do much about exercising body and mind*,* and eating healthy.” (P257)*.

A more pragmatic viewpoint proposed targeting individuals regularly exposed to dementia, such as those affected by dementia or in the early stages of the condition.

*“If you get access to the people who are starting to get warning signs*,* then that to me would be really valuable.” (P114)*.

However, there were also suggestions to focus on raising awareness by including those less aware or not exposed to dementia and lacking understanding of the condition.

*“For someone who has very little understanding of dementia and has maybe encountered it for the first time in later years.” (P615)*.

Regardless, the program was proposed to be beneficial for individuals lacking education or medical information, offering them new knowledge. Additionally, there was a proposal to target younger populations before the onset of risk factors.

*“You really want to catch people young in a preventative sense*,* you need to be before the risk factors are in place.” (P34)*.

## Discussion

Our study explored the feasibility and acceptability of the Brain Bootcamp intervention tailored for older adults, with findings indicating a widespread sense of general acceptability and commendation among participants. Although dropout rate was high, program completers described favourable perceptions of the program, expressing a commitment to continue the acquired brain-healthy behaviours derived from the intervention. Furthermore, multifaceted program resources had recurrent usage among participants, albeit with variations in frequency, to instill brain-healthy behaviours. Future directions should consider the barriers identified, including issues of motivation, self-discipline, and the need for ongoing support to better support dementia risk reduction practices.

The program’s overall feasibility and acceptability suggest a positive receptivity among older adults to targeted interventions delivered in the comfort of their homes, at low costs. This realisation not only affirms the viability of community-based, self-directed initiatives [34, 35] but also presents new opportunities for the advancement of public health programs that could be integrated into participants’ daily lives [36]. The emphasis on delivering interventions in the home environment is further aligned with the growing recognition of the importance of ecological validity in health promotion strategies for older adults [37–39], and the socio-ecological model, which suggests an interconnectedness of individual, interpersonal, and environmental factors in shaping health behaviours [40–42]. Our program’s success in leveraging the home environment further resonates with a body of research advocating for interventions that consider and incorporate the everyday contexts of participants [43, 44], which has been identified as a critical factor in the long-term success of public health programs [40].

The recognition of psychological considerations in our results not only suggests the importance of understanding individual motivational dynamics for dementia risk reduction but also prompts a critical examination of behaviour change strategies. Past literature has consistently highlighted the role of motivation in behaviour change [45–48] whilst our results describe the need to address motivational dynamics in health behaviour interventions. Indeed, participants’ appreciation for the program’s reinforcement of existing habits and knowledge suggests a valuable aspect of the intervention. This finding aligns with prior literature which emphasises the importance of building on individuals’ existing knowledge and practices when promoting behaviour change [49, 50]. However, it is important to recognise that this approach may primarily benefit those who are already somewhat prepared to change. The participants who completed the study may have been at advanced stages of readiness, as indicated by interview quotes suggesting that the intervention merely reinforced their existing behaviours. Therefore, while our program effectively taps into positive psychological mechanisms that align with participants’ self-perceptions and facilitates the adoption of brain-healthy behaviours, its applicability to pre-contemplative individuals (those who are not yet ready or willing to change) remains uncertain. Future research should explore tailored strategies that could engage individuals at various stages of the behaviour change spectrum, including those who may require additional support to initiate change.

The internal versus external motivation debate further adds depth to the understanding of participants’ engagement with the program. Our program’s dual ability to inspire behaviour change and to provide a sense of routine and function aligns with self-determination theory [51], which posits that both intrinsic and extrinsic motivation play essential roles in sustaining behaviour change. Research suggests that interventions incorporating routine or habitual elements and provide a sense of structure can positively impact participants’ adherence and outcomes which is reflected in participant appraisal and engagement with our Brain Bootcamp initiative [52, 53]. The individual differences between level of participation in the program (i.e., extent of goals set and goal attainment amongst participants) could be further explained by the role of self-efficacy when governing intention and maintenance of health behaviour change. Greater self-efficacy is linked to increased responsiveness to behavioral intervention and a reduced likelihood of relapse [54], as opposed to lower self-efficacy levels. The latter may encompass participants who were either more or less engaged due to pre-existing lifestyle choices, as well as those who mentioned previous experiences with low motivation levels [55].

Although Brain Bootcamp was developed for participants at different stages of behaviour change and aimed to promote healthier lifestyle choices for varied self-efficacy levels, deeper exploration of salient, related factors would be useful to boost participation in future multidomain brain health programs. This examination will further help determine whether the program has an impact on longer-term change or self-efficacy beliefs towards emerging brain health risk behaviours [56]. Moreover, the reassurance aspect of our intervention highlights the benefits of receiving social support and positive reinforcement in facilitating behaviour change and maintenance [57]. The program’s ability to offer not only motivation for change but also a supportive environment is a strength that can contribute to its sustained effectiveness over time.

The identified demographic variances in goal setting and achievement are consistent with established literature on health behaviour and goal attainment. Corresponding with previous research, females demonstrated a heightened proclivity for establishing and accomplishing goals, particularly in social and cognitive domains [58–60]. This aligns with well-documented trends suggesting that women are generally more proactive in adopting and adhering to health-related behaviours [61, 62]. Our findings also support the idea that individuals born in English-speaking countries show a more robust inclination towards goal setting and suggest the potential influence of cultural and linguistic factors on health-related behaviours [63–66]. Indeed, the success of English-speaking individuals in achieving their goals corresponds with literature emphasising the facilitative role of language proficiency and cultural alignment in health-related goal accomplishment [67–72], and indicates that future work needs to address language disparities.

Educational attainment emerged as a significant determinant and reflected the well-established link between higher education levels and increased health-conscious behaviours [73–76]. Our finding that individuals with lower educational attainment set more physical activity goals but fewer social and cognitive goals suggest the need for risk reduction interventions to be tailored to diverse educational backgrounds. Future research needs to understand the associations of cultural, linguistic, and educational factors to better elucidate the mechanisms underpinning these observed patterns and to inform the development of more tailored and effective interventions promoting health-related goal accomplishment.

### Strengths and limitations

This study successfully piloted the Brain Bootcamp program among older adults in Australia. Our research used a robust methodology with a large sample size that represented diverse demographics as well as variations in individual gender, locality and education levels. The mixed-method design further enhances the depth of the findings and provides a relatively comprehensive understanding of the program’s acceptability among participants, particularly with over 150 semi-structured interviews. However, although this study adopted a single-group design due to resource limitations, it is acknowledged that a randomised controlled trial format, incorporating either a usual care control or a waitlist control group, would provide a more robust evaluation of the intervention’s efficacy. Future research should consider matching participants with comparable individuals who did not receive the intervention to gain clearer insights into its effects. Additionally, implementing longitudinal observation over a longer period of time would be beneficial in assessing the sustainability of behaviour changes over time and could enable comparisons with other studies with similar follow-up periods. Such subsequent studies can support our understanding of the longer-term impact of these multidomain programs.

Several limitations warrant further consideration, with a strong emphasis on the high attrition rate reported for program completion as well as with individuals who provided qualitative feedback. The high attrition rate observed in the Brain Bootcamp program introduces significant challenges in drawing conclusive insights concerning the feasibility and acceptability of the intervention. This high dropout rate suggests that those who completed the program likely represent a highly motivated and potentially unrepresentative subset of participants. As a result, our findings may not be generalisable to a broader population. In terms of feasibility, the attrition also indicates that while the program was manageable for those who persevered, it may not be practical or sustainable for a wider audience and therefore raises concerns about its scalability in real-world settings. Similarly, the high dropout rate compromises the assessment of acceptability, as the positive feedback from the remaining participants may not accurately reflect the experiences of those who found the program too demanding or disengaging to continue. This suggests that the program may only be appropriate for a very specific group and as such, our conclusions about its impact are carefully framed to acknowledge that it may not be feasible or acceptable for a more diverse or typical population. Indeed, our study lacked representation in terms of cultural and socioeconomic diversity, and relying on retrospective accounts of participants’ behaviour change and goals set or achieved potentially impacts the reliability of results for different demographic groups. The untailored approach and focus on community-dwelling older adults aged 65 years and over further limited the broader applicability of the findings. Nonetheless, our study’s findings raised the need for more targeted strategies, adaptable materials for individuals with pre-existing conditions, and increased communication and support from the research team. Furthermore, participants suggested a more accountable and guided approach to better enhance motivation and self-discipline in future dementia prevention programs, which could include monitoring of lifestyle behaviours and continuous feedback.

Examining these factors (i.e., cognitive ability, motivational levels, personal preferences) in future studies can provide a more complete understanding of the observed positive receptivity. Additionally, exploring variations in receptivity and acceptability among different demographic groups may offer insights into tailoring interventions for diverse populations [77]. There is also a need for prolonged intervention periods to enhance statistical robustness. Future research should leverage our insights to conduct long-term, comprehensive studies that not only encompass a broader age range but also incorporate a more exhaustive examination of various health domains and adopting more robust objective measures of program item use.

## Conclusion

In conclusion, the Brain Bootcamp program’s successful implementation among older Australian adults highlights its feasibility and adaptability. Our findings emphasise the importance of targeted strategies and enhanced communication in future research to improve inclusivity and effectiveness. We have identified a preliminary foundation for fostering brain health in older adults within their homes and offer insights that may contribute to the development of future community-based dementia risk reduction initiatives.

## Electronic supplementary material

Below is the link to the electronic supplementary material.

Supplementary Material 1

## Data Availability

Data is provided within the manuscript or supplementary information files.

## Declarations

### Clinical trial registration

Registered with Australian New Zealand Clinical Trials Registry (ACTRN12621000165886) on 17 February 2021.
