# Supplementary material for: Acceptability and fidelity of the multidomain ‘Brain Bootcamp’ dementia risk reduction program: a mixed-methods approach
Source: BMC Public Health. 2025 Feb 14;25:619. doi: 10.1186/s12889-025-21641-7 (PMC11829373; doi:10.1186/s12889-025-21641-7)
Supplement: Supplementary file 1 — Supplementary Material 1 [file 12889_2025_21641_MOESM1_ESM.docx]

**Appendix**

**I. Survey**

**Indicate your level of agreement with the below statements.**

|  | **Strongly Agree** | **Somewhat Agree** | **Neither agree nor disagree** | **Somewhat Disagree** | **Strongly Disagree** |
| --- | --- | --- | --- | --- | --- |
| **Brain Bootcamp increased my awareness about dementia risk factors** |  |  |  |  |  |
| **I learnt a lot about brain healthy behaviours.** |  |  |  |  |  |
| **I found my personalised brain health profile useful.** |  |  |  |  |  |
| **I made goals to support my brain health (e.g. walking 6,000 steps)** |  |  |  |  |  |
| **I was provided with the right amount of resources to change my lifestyle.** |  |  |  |  |  |
| **Brain Bootcamp improved my brain health.** |  |  |  |  |  |
| **I will continue to maintain my brain healthy behaviours.** |  |  |  |  |  |
| **Overall, I was satisfied with the Brain Bootcamp initiative.** |  |  |  |  |  |

**Please indicate your usage with the items in the box**

|  | **<Once a month** | **<Once a week** | **≥ Once a week** | **Everyday (or close to)** | **Did not use** |
| --- | --- | --- | --- | --- | --- |
| **Social calendar** |  |  |  |  |  |
| **Olive oil and balsamic vinegar** |  |  |  |  |  |
| **Brain activity cards** |  |  |  |  |  |
| **Pedometer** |  |  |  |  |  |
| **Education booklet** |  |  |  |  |  |

**Did you set goals for the below domains? If yes, what were these goals?**

|  | **Yes** | **No** | **Prefer not to answer** |
| --- | --- | --- | --- |
| **Physical activity** |  |  |  |
| **Social activity** |  |  |  |
| **Cognitive activity** |  |  |  |
| **Diet** |  |  |  |

**Did you achieve the goals that you set for the below domains?**

|  | **Yes** | **No** |
| --- | --- | --- |
| **Physical activity** |  |  |
| **Social activity** |  |  |
| **Cognitive activity** |  |  |
| **Diet** |  |  |

**II. Interview guide**

**Questions**

- How did you find out about the campaign?
- Why did you decide to register for Brain Bootcamp?
- Please tell us what you liked most about the campaign? Why?
- Please tell us what you did not like about the program? Why?
- Did our initiative make a difference for you? How so?
- For whom, and in what situations do you think this initiative can best make a difference?
- How did the program support your awareness of dementia risk factors?
- Did it help you with changing your current lifestyle behaviours – if so, how?
- We used different strategies for this program. These are your personalised brain health profile, the items in the box and any goals you may have set.
- What is your opinion on the brain health risk profile?
- What is your opinion on the Brain Bootcamp box?
  - Please tell us what you liked most about the box?
  - Please tell us what you disliked most about the box?
- Have you used any of the items?
  - If so, which ones and why? How frequently did you use it?
  - If not, which ones and why not?

We note in your follow-up survey that you managed to set some goals. Can you tell us about how you decided on these specific goals? Were there any barriers for you in achieving your goals?

- Please tell us what changes could be made to improve Brain Bootcamp?
- What is needed for the initiative to work elsewhere, e.g., in aged care, for rural location, for participants in culturally and linguistically diverse backgrounds?
- How can the team further support future participants?
